# Relative Estimation of Water Content for Flat-Type Inductive-Based Oil Palm Fruit Maturity Sensor

**DOI:** 10.3390/s17010052

**Published:** 2016-12-28

**Authors:** Norhisam Misron, Nor Aziana Aliteh, Noor Hasmiza Harun, Kunihisa Tashiro, Toshiro Sato, Hiroyuki Wakiwaka

**Affiliations:** 1 Faculty of Engineering, Universiti Putra Malaysia, 43400 Serdang, Selangor, Malaysia; aziana.teh@gmail.com; 2 Institute of Advance Technology (ITMA), Universiti Putra Malaysia, 43400 Serdang, Selangor, Malaysia; 3 Medical Engineering Section, Universiti Kuala Lumpur-British Malaysia Institute, Batu 8, Jalan Sg Pusu, 53100 Gombak, Selangor, Malaysia; noorhasmiza@unikl.edu.my; 4 Faculty of Engineering, Shinshu University, Wakasato 4-17-1, Nagano 380-8553, Japan; tashiro@shinshu-u.ac.jp (K.T.); labyam1@shinshu-u.ac.jp (T.S.); wakiwak@shinshu-u.ac.jp (H.W.)

**Keywords:** inductive concept, air coil, resonance frequency, oil palm, maturity classification, moisture content

## Abstract

The paper aims to study the sensor that identifies the maturity of oil palm fruit bunches by using a flat-type inductive concept based on a resonant frequency technique. Conventionally, a human grader is used to inspect the ripeness of the oil palm fresh fruit bunch (FFB) which can be inconsistent and inaccurate. There are various new methods that are proposed with the intention to grade the ripeness of the oil palm FFB, but none has taken the inductive concept. In this study, the resonance frequency of the air coil is investigated. Samples of oil palm FFB are tested with frequencies ranging from 20 Hz to 10 MHz and the results obtained show a linear relationship between the graph of the resonance frequency (MHz) against time (Weeks). It is observed that the resonance frequencies obtained for Week 10 (pre-mature) and Week 18 (mature) are around 8.5 MHz and 9.8 MHz, respectively. These results are compared with the percentage of the moisture content. Hence, the inductive method of the oil palm fruit maturity sensor can be used to detect the change in water content for ripeness detection of the oil palm FFB.

## 1. Introduction

The *Elaeis guineensis* species of oil palm tree are the most common type used for palm oil production. Oil palm fruit will undergo a process through crude palm oil milling to get the crude palm oil for various products. The ripeness of the oil palm is commonly determined based on visual inspection which is manually done by a human grader. The manual grading proposed by the Malaysian Palm Oil Board (MPOB) is used as the main reference during the inspection for harvesting the oil palm fruit bunches. The inspections are based on the surface’s visual color of the oil palm fruit and the number of fruit loose from the bunch [1]. Furthermore, it is important to pluck the oil palm fresh fruit bunch (FFB) at the right time, combined with the accurate ripeness assessments of the oil palm fruit, in order to maintain the optimum quality of palm oil.

To increase the efficiency as well as the accuracy of the oil palm FFB ripeness detection, researchers proposed a new non-destructive detection that can replace the conventional visual method. Various methods for oil palm fruit maturity detection have been developed and tested.

The color vision system is one of the popular methods used for this application. Osama et al. [2] proposed a method of categorizing oil palm FFB maturity through the use of a portable four-band system. The classification system uses a portable and active optical sensor which consists of four spectral bands. The classification of the sensor uses an advanced digital camera and a computer set that is required for the data analysis [2,3]. However, this method requires continuous light intensity monitoring during the image acquisition process for it to be accurately determined by the computer.

The next method is done by using a microwave moisture sensor which utilizes the moisture content of the oil palm fruitlets. Yeow et al. [3,4] proposed an application of the microwave moisture sensor for the identification of the ripeness of oil palm fruit where the content of moisture ranged from 30% to 80% on the basis of wet weight as the main objective of the study. The fabricated sensor operates at the frequency between 1 GHz and 5 GHz, and the overall system is comprised of the fabricated sensor and a vector network analyzer (VNA) that is computer-controlled. The procedure to conduct the measurement is complicated and time-consuming, and the equipment must be used indoors.

Shariffudin et al. [5] presented a monitoring system for oil palm fruit development and ripeness using magnetic resonance imaging (MRI) along with bulk nuclear magnetic resonance (NMR). The proposed monitoring system focused on tracking the development of intact fruit. The entire measurements of spin-spin relaxation times (T2 values) were conducted at the level of 2.35 Tesla and at 200 °C. This method requires skilled personnel to operate complicated and expensive machines, which limit it to indoor testing.

Non-destructive near-infrared (NIR) spectroscopy uses two spectrometers to examine the oil palm fruit with different modes and their chemical content is analyzed using the partial least square regression (PLSR) model [6]. This method again requires indoor testing, in addition to the complicated sample preparation as well as the expensive equipment.

This paper is about a new grading method which uses a simple inductive technique, where the focus is based on the resonance frequency of the air coil as well as the change in the air coil’s stray capacitance.

## 2. Design Basic Principles

### 2.1. Electrical Diagram

**Figure 1:**
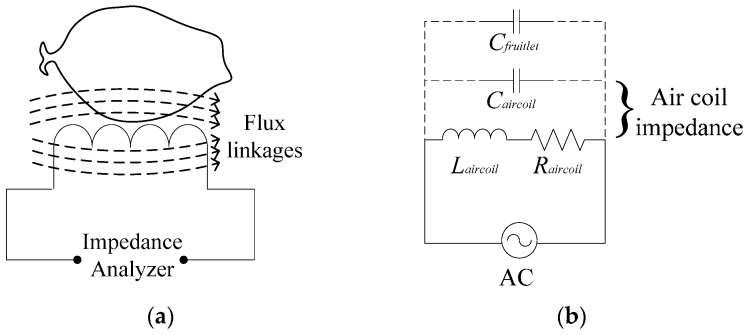
(**a**) Single flat-type shape air coil electrical diagram; (**b**) Series RLC circuit representation.

The single flat-type coil representation is shown in Figure 1a. The flux linkages flow through the fruit sample and the inductance of the air coil is measured by using an impedance analyzer. The whole system of the inductive oil palm fruit sensor can be further explained as illustrated in Figure 1b. This inductive oil palm fruit sensor requires operating at the high-frequency response and thus the effect of inductance is very high. The value of the capacitance is predicted from the value of the stray capacitance which came from both the air coil and fruitlet samples.

**Figure 2:**
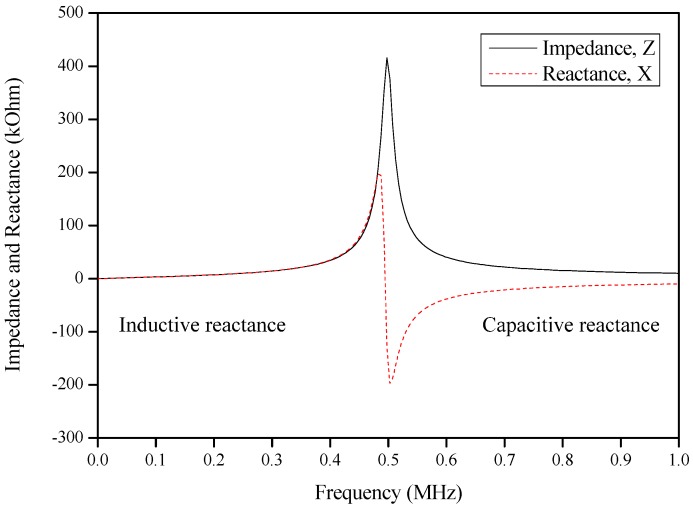
Example of the air core inductor impedance and reactance.

For each non-ideal inductor, it has a special frequency point named as a natural frequency where the impedance of the circuit is dominated by inductive properties at low frequency, while at high frequency it is dominated by capacitive properties. At the intermediate point in between two properties, the impedance transition becomes purely resistive. This is the resonance frequency of an RLC circuit, also known as a resonant circuit, in radians per second and every non-ideal inductor has it. This resonance point is called the self-resonance frequency of the inductor. The self-resonance frequency is where the inductor will start to behave like a capacitor beyond the self-resonance point. The resonant frequency is the frequency where the inductive reactance and capacitive reactance are the same and cancel one another. This response is shown in Figure 2. The reactance, up to the resonant frequency, is positive, but beyond resonance, the reactance becomes negative. From basic circuit theory, it is known that a negative reactance is associated with a capacitor. Therefore, above the self-resonance frequency (SRF), the inductor behaves like a capacitor.

The resonant frequency of the inductive oil palm fruit sensor is assumed to have a constant by considering the effects of both the air coil structure and coil casing. Therefore, it can be derived as follows:

(1) $$f_{r}=\frac{1}{2\pi \sqrt{LC}}$$

where *f_r_*, *L* and *C* are the resonant frequency (Hz), inductance (H) and capacitance (F), respectively.

The values of the inductance and capacitance are based on the estimation calculation of the air coil’s structure. Through this empirical analysis, the value of the capacitance is estimated based on the existence of stray capacitance in the air coil. The capacitance value of this flat-type air coil uses the simplified formula which estimates the capacitance to determine the self-resonance frequency of the inductor [7].

This proposed inductive concept of the oil palm fruit sensor involves the moisture content which is also related to the changes in the permittivity of the fruitlet. Since the oil palm fruitlet is a non-conducting material, the permeability value of the fruitlet is extremely small. It is identified that the permeability value of water is 1.2566270 × 10^−6^ Hm^−1^ and the permeability of air, *µ*_0_, is 1.256637061 × 10^−6^ Hm^−1^. As for the capacitance, the permittivity value for air, *ε*_0_, is 8.854 × 10 ^−12^ Fm^−1^, while the relative permittivity of water and oil are 80 and 2~3, respectively [8].

### 2.2. Sensor’s Structure

**Figure 3:**
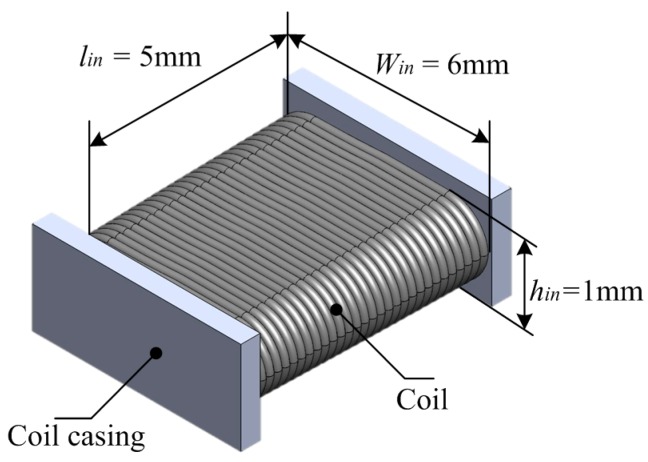
Flat-type air coil 3D structure.

The flat-type oil palm fruit sensor is rectangular in shape as shown in Figure 3. The main parameters of the air coil consist of the height (inner height, *h*_in_ = 1 mm), width (inner width, *w*_in_ = 6 mm) and length (inner length, *l_in_* = 5 mm) of the sensor. The sensor’s fabrication uses a plastic called Perspex which is a non-conducting material that minimizes the flux disturbance in the sensor.

With the coil wire diameter *D_o_* of 0.12 mm, the sample of fruitlets from the oil palm fresh fruit bunch is tested with a frequency ranging between 20 Hz to 10 MHz for a total duration of 16 weeks. The air coil’s number of turns is fixed at 170 turns. The set-up and parameters shown in Table 1 are constant throughout the experiment.

### 2.3. Resonance Characteristics

**Figure 4:**
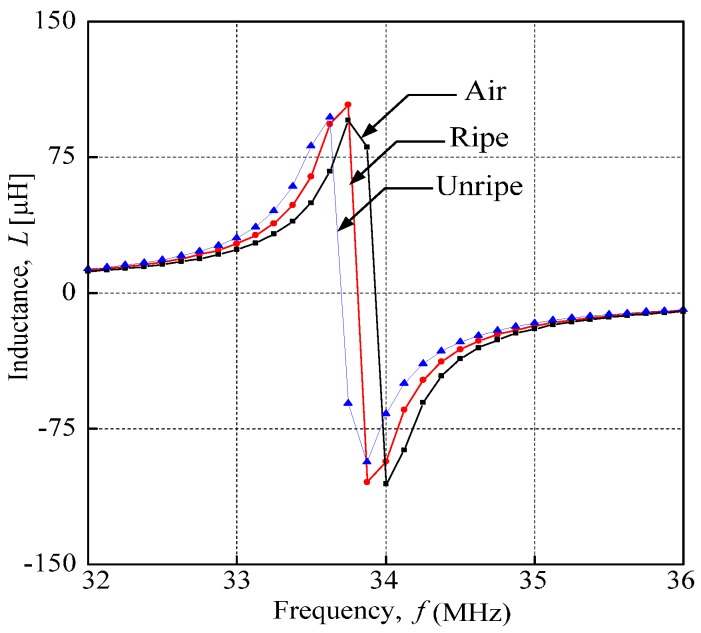
Oil palm fruit sensor inductance characteristics for air, ripe and unripe fruits.

Through analyzing the inductance characteristics for air, and ripe and unripe fruitlets, the graph shows different resonance frequency values with similar curves for all samples. The resonance frequency from this experiment is identified where the inductance is at its maximum value. From Figure 4, the inductance characteristics of each sample are represented by colors; that is, the black line represents air, the red line represents ripe fruit and the blue line represents unripe fruit. The sensor portrays a similar inductance characteristic curve for all samples throughout the experiments.

### 2.4. Fruit Sample

**Figure 5:**
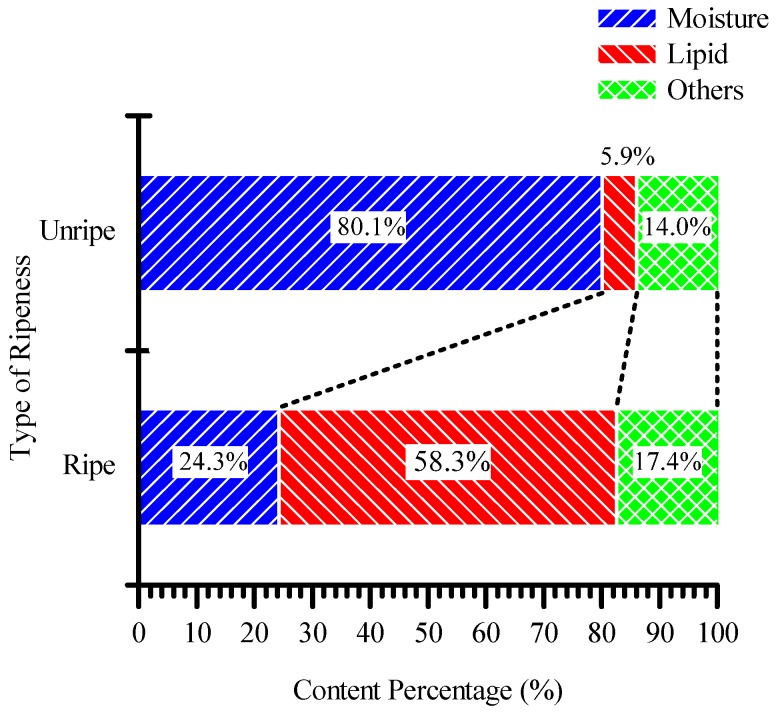
The percentage of content for unripe and ripe oil palm fruit.

Sample selection and the category are standardized throughout the research. Each sample was taken from the same bunch; each sample was measured three times to ensure the consistency of the measurement for each sample. Standard specifications outlined by MPOB are used in sample selection, such as the fruitlet surface’s color and its age. The ripe fruitlet is orange whereas the unripe fruitlet sample is dark purple in color. Unripe fruitlet samples were selected around the seventh weeks after anthesis (WAA) and at 18th WAA for the ripe fruitlet [9]. Each sample was freshly taken from the same oil palm fresh fruit bunch (FFB) on the testing day. The test had to be completed on same day that samples were taken in order to avoid inconsistency and contamination. The characteristics of the samples selected for this research are summarized in Table 2. Figure 5 shows the percentage content of the ripe and unripe oil palm FFB that was tested for its content.

## 3. Mathematical Analysis

### 3.1. Inductance Estimation

Based on the parameters set up in Table 1, inductance for the single flat-type air coil was deduced from the air coil’s formula, proposed in Reference [10]. For a finite length of coil wire, the inductance of the flat-type air coil can be calculated using Nagaoka’s coefficients as in the following equation:

(2) $$L_{aircoil}=C_{nagaoka}\;L_{0}$$

where *L_aircoil_* (H) is the inductance of the air coil, *L*_0_ (H) is the inductance of the ideal inductor and *C_nagaoka_* is the Nagaoka’s coefficient. The formula for the ideal coil *L*_0_ is as follows:

(3) $$L_{0}=\frac{\mu_{o}\pi N^{2}{\left(W_{in}+D_{o}\right)}^{2}}{4\;l_{in}}$$

where *µ*_0_ is the permeability in vacuum (H/m), *N* is number of turns of the coil windings, *W_in_* is the inner width of the air coil (m), *D_o_* is the diameter of the coil wire (m), and *l_in_* is the inner length of the air coil (m). The Nagaoka coefficient can be found by the following equation:

(4) $$C_{nagaoka}=\frac{4}{3\pi }\frac{1}{k^{\prime }}\left[\frac{{k^{\prime }}^{2}}{k^{2}}\left(K-E\right)+E-k\right]$$

(5) $$k=\sqrt{\frac{{\left(W_{in}+D_{o}\right)}^{2}}{{\left(W_{in}+D_{o}\right)}^{2}+{l_{in}}^{2}}}$$

where *k* is the elliptic modulus, *k′* is the complementary elliptic module, *K* is the complete elliptic integral of the first type and *E* is the elliptic integral of the second type. The complete elliptic integral of the first type *K* and the second type *E* are calculated to estimate the value of inductance [10]. The definitions for both *K* and *E* are described by the following equations:

(6) $$K=K\left(k\right)=\int_{0}^{\pi /2}\frac{1}{\sqrt{1-k^{2}\sin^{2}\theta }}\;d\theta$$

(7) $$E=E\left(k\right)=\int_{0}^{\pi /2}\sqrt{1-k^{2}\sin^{2}\theta }\;d\theta$$

Since the calculation of the coefficient requires the estimation of the complete elliptic integral, an approximation is needed for practical and easy approximation calculation. Therefore, C. Hastings’ approach is used for the inductance calculation for this flat-type air coil.

(8) $$K\left(k\right)=\left(1.3862944+0.1119723{k^{\prime }}^{2}+0.0725296{k^{\prime }}^{4}\right)\;\;+\left(\frac{1}{2}+0.1213478{k^{\prime }}^{2}+0.0288729{k^{\prime }}^{4}\right)\ln\left(\frac{1}{{k^{\prime }}^{2}}\right)$$

(9) $$E\left(k\right)=\left(1+0.4630151{k^{\prime }}^{2}+0.1077812{k^{\prime }}^{4}\right)+\left(0.2452727{k^{\prime }}^{2}+0.0412496{k^{\prime }}^{4}\right)\ln\left(\frac{1}{{k^{\prime }}^{2}}\right)$$

### 3.2. Self-Capacitance Estimation

**Figure 6:**
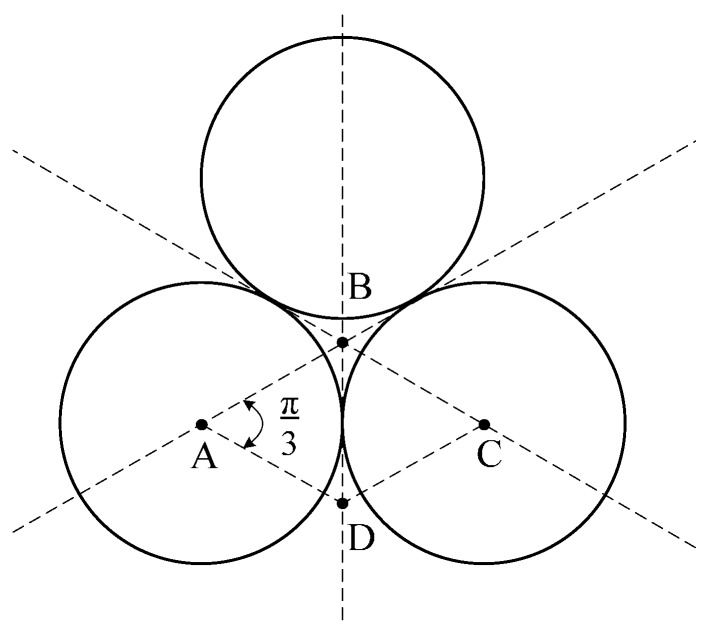
Representation of turn-to-turn capacitance for basic ABCD.

For the air coil’s self-capacitance, the turn-to-turn capacitance is formed from the basic cell shown in Figure 6. It is observed that two adjacent turns of different layers and two adjacent turns of the same layer for the basic cell are identical. Thus, the winding’s inner part can be divided into identical basic cells.

The total stray capacitances of a single basic cell of ABCD from Figure 6 can be calculated using the simplified formula by considering the turn-to-air gap capacitance, the turn-to-turn capacitance and the turn-to-insulation coating capacitance.

A near-accurate simplifying approach obtained from Reference [7] where *θ* = 0° and the plot of Equations (11) and (12) resulted in the path of the electric field in the air gap becoming zero and the corresponding basic cell elementary capacitance given by Equation (11) remaining constant. Therefore, for a small value of angle *θ*, the basic cell air gap’s elementary capacitance is larger than the series combination of the coating’s elementary capacitance.

Due to the coil’s geometrical symmetry, the electric field lines need to be equally shared between the adjacent conductors. Therefore, by considering two adjacent conductors, the basic cell elementary capacitance *dC* between two opposite corresponding elementary surfaces of the conductor *dS* is given by:

(10) $$dC=\epsilon \frac{dS}{x}\;(F)$$

where *ϵ* = *ϵ*_0_*ϵ_r_* is the permittivity of the medium with *ϵ_r_* = 5.1 for enamel, and *x* is the length of the electric field line connecting two elementary surfaces. Each basic cell elementary surface location can be described by an angular coordinate, *θ*. Hence, the basic cell elementary capacitance *dC* depends on the angular coordinate, *θ*.

The insulating coating equivalent capacitance in the basic cell *C_ttc_* is given by:

(11) $$C_{ttc}=\epsilon_{0}\epsilon_{r}\frac{\pi W_{in}\theta }{\ln\frac{D_{o}}{D_{c}}}\;(F)$$

From the geometrical consideration, the length of the assumed path and the elementary surface of the wire which includes the coating are in the form of the elementary ring of the length, $\pi W_{in}$. The air gap capacitance as the elementary capacitance per unit angle *C_ttg_* is:

(12) $$C_{ttg}=\epsilon_{0}\pi W_{in}\left[\cot\left(\frac{\theta }{2}\right)\cot\left(\frac{\pi }{12}\right)\right]\;(F)$$

Angle θ* corresponds to the crossing point at which Equations (11) and (12) can be equated, which yields:

(13) $$\theta^{*}=\cos^{-1}\left(1-\frac{\ln\frac{D_{o}}{D_{c}}}{\epsilon_{r}}\right)$$

Therefore, the total capacitance of the basic cell is the parallel combination of the capacitance, *C_tt_*:

(14) $$C_{tt}=C_{ttc}+C_{ttg}=\epsilon_{0}\pi W_{in}\left[\frac{\epsilon_{r}\theta^{*}}{\ln\frac{D_{o}}{D_{c}}}+\cot\left(\frac{\theta^{*}}{2}\right)-\cot\left(\frac{\pi }{12}\right)\right]\;(F)$$

Assuming that the overall stray capacitance of the coil with N turns is in sequence, it converges to:

(15) $$C_{s}=1.83C_{tt}\;(F)\;\mathrm{for}\;N\geq 10$$

## 4. Results and Discussions

### 4.1. Calculated Results

Based on specification in Table 1 and Figure 2, the value of the inductance, the self-capacitance and the resonance frequency is calculated.

The inductance value is calculated with Equation (2). The value of the Nagaoka coefficient, *C_nagaoka_*, is 0.642941286 as acquired by Equation (4) together with Equations (5)–(9). The value of the ideal coil *L*_0_, obtained from Equation (3), is 213.7 μH. Therefore, the estimated inductance of the flat-type air coil *L_aircoil_* is 137.4 μH.

The self-capacitance approximation of this flat-type air coil is obtained from the total capacitance of the basic cell *C_tt_* from Equation (14), which is 0.920 pF, with the angular coordinate of the intersection angle for Equations (11) and (12), which is θ* = 0.4326 rad or 24.79°. From Equation (15) we can obtain the approximate value of the stray capacitance, *C_s_* = 1.68397 nF. From both values of *L_aircoil_* and *C_s_*, we can predict that the resonance frequency from Equation (1) is 10.5 MHz.

It is expected that the calculated value of the resonance frequency exceeds the experimental value. The calculation does not include the capacitance value of the fruitlet and it just considers the capacitance of the air coil only. Furthermore, it is also noted that the approximation does have an error percentage that affects the calculated resonance frequency to be higher than the actual value.

### 4.2. Field Testing Results and Analysis

**Figure 7:**
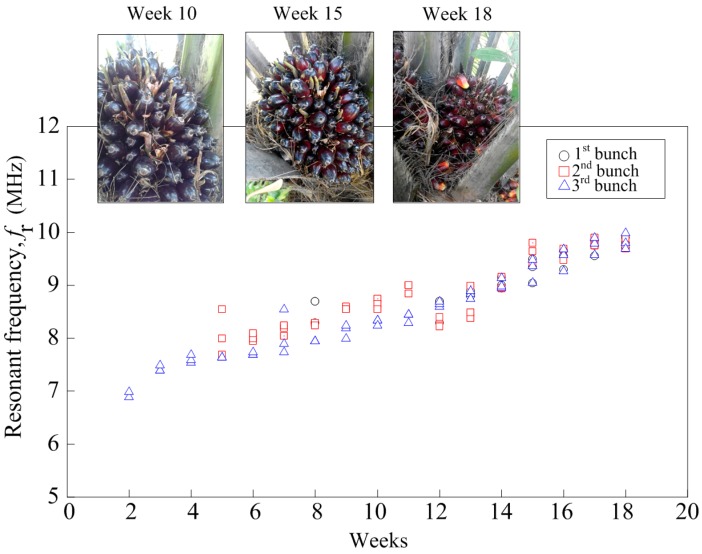
Oil palm fruit sensor frequency characteristics.

Figure 7 shows the results from three bunches of the oil palm FFB at different levels of maturity according to the number of weeks. All graphs show a similar curve where the resonant frequency increases as the week of the testing increase. The field test was conducted simultaneously on three different fruit bunches until they fully ripened. The number of weeks it took for each bunch to reach its optimum maturity differed for each bunch, as these bunches were initially at different stages of ripeness. From Figure 7, the blue-colored symbol represents the bunch that was predicted to be at eight weeks of anthesis when the field testing started and it ended after 10 weeks as the fruitlet started to loosen from the bunch. The red-colored symbol represents the bunch predicted to be at the age of four weeks of anthesis while the black-colored symbol represents the bunch at two weeks of anthesis. All three bunches provided a similar pattern throughout the testing period and the range of the resonant frequencies for all these bunches was recorded within 6.3 MHz to 9.9 MHz.

The resonant frequencies of these three bunches were recorded at 9.8 MHz to 9.9 MHz when the fruitlet started to loosen from the bunch and the color of the mesocarp turned to yellowish-red as described in the grading manual published by the Malaysian Palm Oil Board. Figure 7 also shows the picture of the fruit bunches at different levels of maturity corresponding to the weeks the field testing was conducted. The oil palm fruit bunches is predicted to be at 10th WAA, 15th WAA and 18th WAA, respectively. The prediction was based on the week when the fruitlet started to loosen from the bunch and was assigned as 18th WAA.

In order to find the relationship between the maturity of the oil palm fruitlets and the moisture content, we observed the value of capacitance which affects the resonance frequency from the frequency stated in Equation (1).

**Figure 8:**
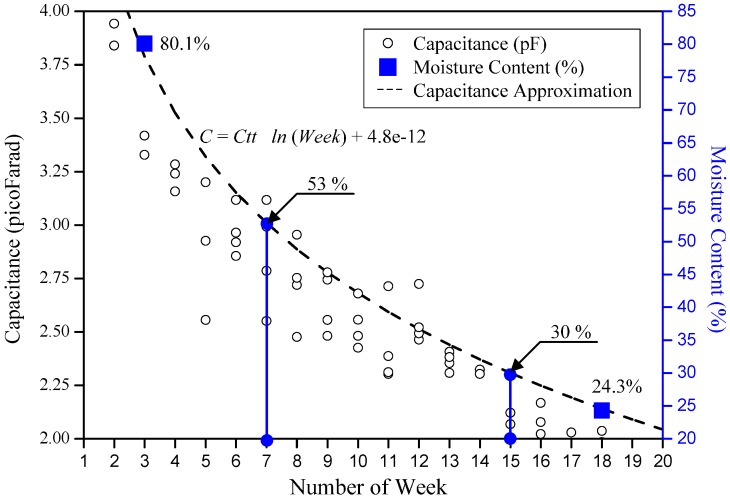
Graph of capacitance and moisture content of the fruitlet against week.

Based on the measured resonance frequency obtained, a graph of capacitance and moisture content against the week is plotted and the significance of the relationship is modeled. An approximation line is plotted and the relation between the moisture content is approximated between the number of weeks and the capacitance. The capacitance values are obtained with Equation (1) using *L_aircoil_* = 137.4 μH as calculated from Equation (2) and the resonance frequency obtained through field measurement. It is observed that as the number of weeks progressed, the capacitance value obtained through the measured resonance decreased. This is due to the loss of moisture in the fruitlet and the increase in lipid content as the fruitlet in the fruit bunches ripened. Referring to the data obtained from Figure 5, the percentage of moisture content for unripe and ripe oil palm fruitlets is marked near the approximation line as shown in Figure 8.

It is deduced that the approximation equation capacitance value, C (F) for respective weeks of the curve is as follows:

(16) $$C=-C_{tt}\;\ln\left(Week\right)+4.8\times 10^{-12}$$

where *C_tt_* is 0.920 pF, Week represents the weeks after anthesis (WAA) of the FFB, followed by the experimental correction for the capacitance approximation curve. This equation is applied only for the moisture content range of 20% to 85%, as a value beyond this range is not possible.

Therefore, from the approximate moisture content of the unripe fruitlet curve, the unripe fruitlet with an 80.1% moisture content, from data in Figure 5, falls near Week 3, and the ripe fruitlet with a 24.3% moisture content falls near Week 18, as shown in Figure 8.

From the graph obtained from Figure 8, the relationship between the capacitance from the resonance and the moisture content percentage can be predicted using the equation below:

(17) $$Percentage\;of\;Moisture\;Content=-MC_{ripe}\;\ln\left(Week\right)+111$$

where *MC_ripe_* is the moisture content of the ripe fruitlet which is approximately 30% [3] and Week refers to the weeks after anthesis (WAA), followed by the experimental correction value for the moisture content approximation equation.

The maturity process of the oil palm FFB starts from the seventh WAA until the 15th WAA [1]. From Figure 8, it is approximated that at the seventh WAA, the water content of the fruitlet of the FFB is estimated at around 53%, and it further decreased at the 15th WAA to 30% which is the recommended time for harvesting [1].

The unripe sample is stated to be selected before the 12th WAA with the initial 80.1% water content, which is coinciding with the estimation curve around the third WAA, which is a plausible estimation. For the ripe sample, it is found that 24.3% moisture content is overlapping the estimation curve at the 18th WAA, which is plausible as the ripe fruitlet is estimated to be of age between the 16th WAA to the 20th WAA [1].

## 5. Conclusions

The flat-type oil palm maturity sensor functions as a device to detect the maturity of the oil palm fruitlet which corresponds with the increase of the measured resonance frequency. It is observed that the ready-to-harvest fruitlet on Week 18 obtains a frequency of 9.8 MHz in comparison to the frequency of 8.5 MHz at Week 10. Further analysis shows that the stray capacitance of the sensor changes as the fruit matures in relation with the moisture content of the fruit. Through the estimation curve, the moisture content of the fruitlet decreases in a negative logarithm function. The experimental correction value in the estimation curve is due to the shape, position and experimental setup for that particular measurement. Thus, there is a potential for this inductive concept to be developed as an oil palm fruit maturity sensor.

## Figures and Tables

**Table 1:** Specifications for field testing experimental setup.

Parameter/Part	Value/Type
Type of Measurement Setup	Series (L_s_–R_s_)
Voltage (mV)	500
Frequency (MHz)	0.02–10
Sweep (points)	200
Coil wire diameter, *D_o_* (mm)	0.12
Number of turns, N	170

**Table 2:** Characteristics of the selected samples.

Category	Surface Color	Age (WAA)
Unripe	Dark purple	After 7
Ripe	Red orange	18–21
